# Nondestructive quality assessment and maturity classification of loquats based on hyperspectral imaging

**DOI:** 10.1038/s41598-023-40553-3

**Published:** 2023-08-14

**Authors:** Shunan Feng, Jing Shang, Tao Tan, Qingchun Wen, Qinglong Meng

**Affiliations:** 1 Food and Pharmaceutical Engineering Institute, Guiyang University, Guiyang, 550005 China; 2 Research Center of Nondestructive Testing for Agricultural Products of Guizhou Province, Guiyang, 550005 China

**Keywords:** Imaging techniques, Near-infrared spectroscopy

## Abstract

The traditional method for assessing the quality and maturity of loquats has disadvantages such as destructive sampling and being time-consuming. In this study, hyperspectral imaging technology was used to nondestructively predict and visualise the colour, firmness, and soluble solids content (SSC) of loquats and discriminate maturity. On comparison of the performance of different feature variables selection methods and the calibration models, the results indicated that the multiple linear regression (MLR) models combined with the competitive adaptive reweighting algorithm (CARS) yielded the best prediction performance for loquat quality. Particularly, CARS-MLR models with optimal prediction performance were obtained for the colour (*R*^2^_P_ = 0.96, RMSEP = 0.45, RPD = 5.38), firmness (*R*^2^_P_ = 0.87, RMSEP = 0.23, RPD = 2.81), and SSC (*R*^2^_P_ = 0.84, RMSEP = 0.51, RPD = 2.54). Subsequently, distribution maps of the colour, firmness, and SSC of loquats were obtained based on the optimal CARS-MLR models combined with pseudo-colour technology. Finally, on comparison of different classification models for loquat maturity, the partial least square discrimination analysis model demonstrated the best performance, with classification accuracies of 98.19% and 97.99% for calibration and prediction sets, respectively. This study demonstrated that the hyperspectral imaging technique is promising for loquat quality assessment and maturity classification.

## Introduction

Loquat (*Eriobotrya japonica* Lindl.) is an evergreen fruit tree of the Rosaceae family, and its fruit is used as a dual-purpose medicine and food that has been cultivated in China for more than 2000 years^1^. It is used for clearing the pharynx, moistening the lungs, alleviating cough, and lowering phlegm^2^. The ripening pattern of loquats is similar to that of climacteric fruits. If harvested very early, it will have hard flesh and a bland flavour. As loquats have an active postharvest physiological metabolism, they are susceptible to water and nutrient loss and rot if harvested late^3,4^. Fruit quality has a direct impact on its commercial value. Colour, firmness, and soluble solid content (SSC) are important characteristics of loquats and are key parameters for evaluating their taste and maturity^5^. Therefore, the detection of postharvest loquats is crucial.

However, traditional determination methods have the disadvantage of destructive sampling and are not suitable for online detection. In recent years, hyperspectral imaging (HSI) techniques, which combine two-dimensional image information with one-dimensional spectral information, have been widely used to evaluate fruit quality and maturity. HSI has been used to determine multiple indicators (SSC, firmness, etc*.*) of fruits, including plums^6^, sweet cherries^7^, pears^8^, peaches^9^, and melons^10^. Extensive studies have been conducted to predict quality and ripeness of fruits. Wei et al*.*^11^ used HSI to classify ripeness and predict the firmness of persimmons. Munera et al*.*^12^ used the index of internal quality and maturity to assess the internal physicochemical attributes and sensory perception of ‘Big Top’ and ‘Magique’ nectarines. The ratio of total soluble solids (TSS) to titratable acidity (TA) was used as a pineapple ripeness index to analyse the effects of transmittance short-wavelength near-infrared spectroscopy and reflectance near-infrared hyperspectral imaging on the prediction of pineapple ripeness using the same procedure and model, respectively^13^. Benelli et al*.*^14^ investigated the potential of using HSI directly in the field through proximal measurements under natural light conditions to predict the harvest time of ‘Sangiovese’ red grape. They split grape samples into two classes based on the reference value of SSC and established models to predict SSC and recognise the maturity stages, respectively. Zhang et al*.*^15^ combined HSI with support vector machine (SVM) to evaluate strawberry ripeness. The results indicated that the SVM model performed the best, with classification accuracy of over 85%.

Furthermore, considerable attention has been given to visualise quality of fruits. Teerachaichayut et al*.*^16^ applied HSI to perform nondestructive detection and visual analysis of TSS and TA and calculated TSS/TA as a measure of the maturity index in intact limes. The predictive distribution maps of TSS, TA and TSS/TA were generated by inputting the feature bands of each pixel into optimal models. Li et al*.*^17^ realised the visualization of SSC and pH based on a colour scale in cherry fruits. Chu et al*.*^18^ created the visualization maps for banana quality parameters using machine learning algorithm. The results indicated that the hyperspectral imaging is a useful tool to assess the quality of bananas. Additionally, due to the complexities involved in processing hyperspectral data and the inherent limitations of computer hardware capabilities, it is essential to select feature wavelengths instead of using full wavelengths to achieve similar precision in the operation. Zhang et al*.*^19^ established partial least squares regression (PLSR) model for predicting caffeine content of coffee beans based on full wavelengths and feature wavelengths using HSI, respectively. The overall results indicated that, similar to PLSR models built on full wavelengths, all PLSR models based on feature wavelengths demonstrated robust performance. Li et al*.*^20^ developed rapid and non-destructive models for detecting anthocyanin content in mulberry fruit using HSI, based on both full bands and feature variables, respectively. The results indicated that the models based on feature variables demonstrated superior performance compared to those using full bands. Sharma et al*.*^21^ applied HSI to classify the ripening stages and predict the dry matter content of durian pulp. A comparison was conducted between the models using full wavelengths and feature wavelengths. The results indicated that the model based on full wavelengths showed comparable performance to the model based on feature wavelengths in maturity classification, while the model based on feature wavelengths achieved better results in predicting dry matter. Most of the above studies have confirmed the feasibility of fruit quality prediction and maturity classification using hyperspectral imaging, and it is crucial to choose feature variables for modelling during data processing. Nevertheless, little research has reported the utility of HSI technology to predict and visualise the colour, firmness, and SSC of loquats and discriminate maturity.

This study aimed to explore the feasibility of determining and visualising the colour, firmness, and SSC of loquats and discriminating maturity based on HSI. The specific objectives of this study were to (1) compare the performance of different feature variables selection methods including competitive adaptive reweighting algorithm (CARS), genetic algorithms (GA), and successive projections algorithm (SPA); (2) establish and compare calibration models for predicting quality including PLSR, principal components regression (PCR), multiple linear regression (MLR), extreme learning machine (ELM), and back-propagation neural network (BP); (3) visualise the spatial distribution of these quality parameters in loquats; and (4) develop recognition models for discriminating maturity including partial least square discrimination analysis (PLS-DA), simplified K-nearest neighbour (SKNN), and SVM models.

## Methods

### Sample preparation

A total of 649 loquats (transverse diameter: 35–55 mm) without bruises were harvested from the commercial orchards (Loquat Green Planting Demonstration Garden of Kaiyang County) located in Guizhou Province, China, on 7 June 2022. The collectors took the permit, which was required at the time, and obtained the owner’s permission. The selection of loquats was guided by experienced local growers based on visual observation of the external colour, ranging from dark green to dark orange. The samples were transported to the laboratory on the same day as the sampling, at a temperature of 23 ± 2 °C. Before the experiment, the loquat surfaces were wiped and numbered. All methods were performed in accordance with the relevant guidelines and legislation.

**Figure 1:**
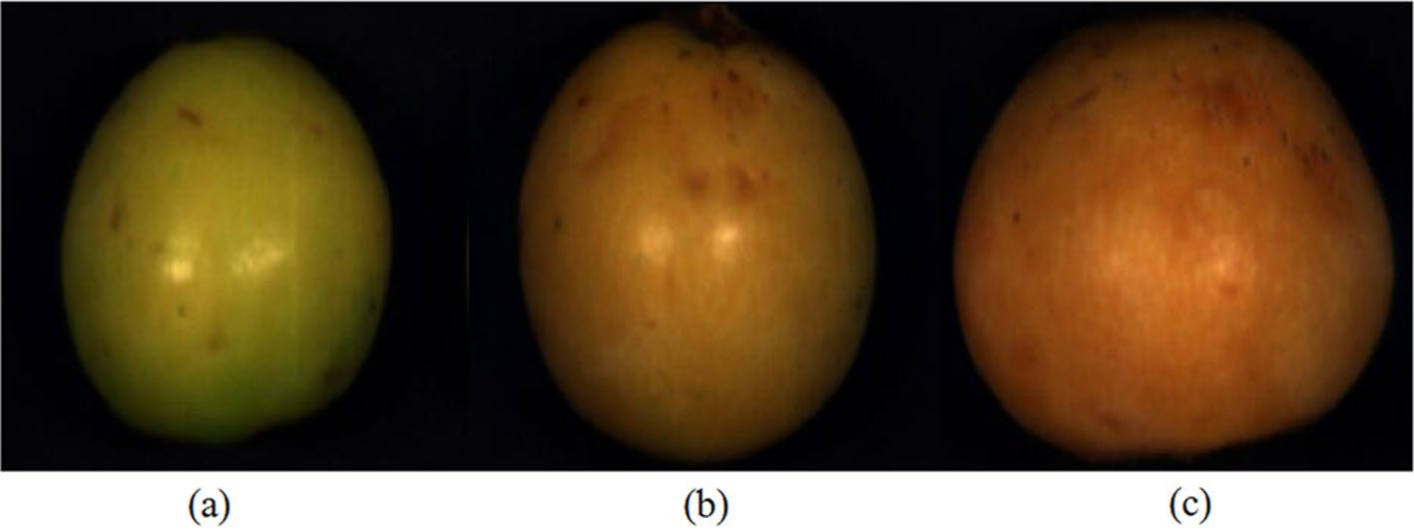
Images of loquat maturity stage I (**a**), maturity stage II (**b**), and maturity stage III (**c**).

Deng et al*.*^22^ found a significant or highly significant correlation between the colour a* value and loquat quality. On this basis, the 649 samples were divided into three maturity stages (stage I: 177, stage II: 331, and stage III: 141) based on the colour a* value. Stage I represented colour a* values less than 8.33, stage II covered colour a* values between 8.33 and 15.41, and stage III encompassed colour a* values greater than 15.41. The images of the three maturity stages are shown in Fig. 1.

To generate adequate variability and broaden the predictive range of colour, firmness and SSC, the samples were divided into four groups for experimentation. Among these samples, 140 were used for predicting loquat colour (stage I: 47, stage II: 63, and stage III: 30), another set of 140 for predicting loquat firmness (stage I: 45, stage II: 53, and stage III: 42), and 120 for predicting loquat SSC (stage I: 25, stage II: 65, and stage III: 30). The remaining 249 samples were used to classify loquat maturity (stage I: 60, stage II: 150, and stage III: 39).

### Hyperspectral image acquisition and correction

**Figure 2:**
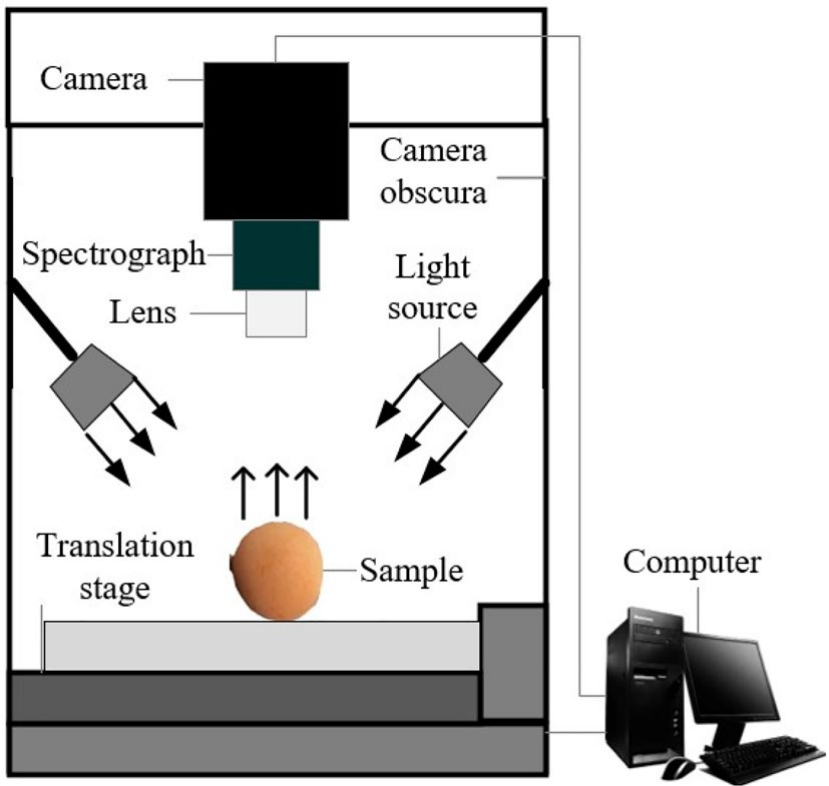
Schematic diagram of the hyperspectral imaging acquisition system.

Hyperspectral images of loquat samples were captured using a hyperspectral imaging system (GaiaFieldF-V10, Jiangsu Dualix Spectral Imaging Technology Co., Ltd). A schematic of the system is shown in Fig. 2. It primarily included a hyperspectral imaging spectrograph (Imspector V10, Spectral Imaging Ltd., Oulu, Finland), CCD camera (Imperx IPX-2 M30, Pixels: 696 × 1313), zoom lens (HSIA-OL23, Focal length: 23 mm), four 200 W halogen light sources (HSIA-LS-T-200 W), transportation plate, dark room (HSIA-T400-IMS), and computer with image acquisition software. The distance from the sample to the lens was 400 mm, and the exposure time of the spectral camera was 12.6 ms. The spectral resolution was 3.5 nm, and the spatial resolution was 0.2 mm/pixels. The spectrograph obtained spectral images covering a wavelength range from 390 to 1030 nm with 256 spectral bands.

When acquiring hyperspectral images each time, four loquats were placed regularly on the sample stage above the displacement platform according to their number^23^. To eliminate the effects of noise and dark current in the CCD camera, the acquired original images were used to correct the black and white images. The correction was performed based on Eq. (1). After the hyperspectral images were corrected, the spectral data from the entire sample area of loquat were extracted by using ENVI 5.4 (ITT Visual Information Solutions, Boulder, CO).

1 $$ I = \frac{I0 - B}{{W - B}} $$

where, *I* is the calibrated image, *I*_0_ is the original image, *B* is the dark reference image, and *W* is the white reference image.

### Reference values for measurement of quality parameters

Following hyperspectral image acquisition, conventional destructive methods were used to measure the reference values for the colour, firmness, and SSC of the loquats. For the determination of colour, a spectrophotometer (Ci7800) was used to measure the colour parameters (L*, a*, and b* values), which were evaluated using colour *e* value calculated based on Eq. (2)^24^. The formula emphasizes the colour contrast in the a* and b* directions, enabling a more effective comparison of colour characteristics among different loquats.

2 $$ e = \frac{1000a*}{{(L* \times b*)}} $$

Firmness was measured using a texture analyser (TA.XT.plus) with a cylindrical puncture probe of 2 mm at a test speed of 3 mm/s. The measurement required the peeling of the loquat around the equator.

The measurements of the SSC were carried out using a digital refractometer (PAL-*α*) in the range 0–85%.

### Data preprocessing and feature variables selection

To improve the accuracy and stability of the model, spectral pre-processing aims to eliminate instrument noise, scattering, and baseline shifts. Standard normal variation (SNV) was used to preprocess the original spectra; it can reduce the effects of surface scattering and light path alterations on diffuse reflection^25^.

Additionally, the hyperspectral data were characterised by redundancy and multicollinearity. To reduce the number of modelling calculations and improve the operational efficiency of the model, the CARS, GA, and SPA were applied to select the feature variables. Variable points with large absolute values of the regression coefficients in the PLSR model established by CARS are selected as the new correction set, and the subset with the smallest root mean square error was obtained after several cycles^26^. The GA simulates the mechanisms of natural selection and genetics and iteratively performs operations to generate a subset of variables^27^. Unlike GA, SPA is a forward feature variables selection method that minimises the collinearity between feature vectors^28^.

### Model building and evaluation

Two commonly used tools for multivariate data analysis, PLSR and PCR models, were developed by combining chemical concentration and preprocessed data, respectively^29^. Subsequently, three feature variables models, namely, MLR, BP, and ELM models, were established based on the selected feature variables. MLR is used to characterise the relationship between spectral data and mass parameters using a linear fitting equation^30^. BP, which is one of the most typical multilayer forward network, is a local optimisation method based on gradient descent^31^. ELM is a high-efficiency single hidden layer feed-forward neural network that can map nonlinear relationships between input and output values^32^.

To evaluate the performances of the prediction models, the determination coefficient of the calibration set (*R*^2^_C_), root mean square error of the calibration set (RMSEC), the determination coefficient of the prediction set (*R*^2^_P_), root mean square error of the prediction set (RMSEP), and residual predictive deviation (RPD) were calculated. Generally, a model that performs well has higher values of *R*^2^_C_, *R*^2^_P_, and RPD and lower values of RMSEC and RMSEP. The model performs poorly when the RPD is lower than 1.5, whereas an RPD between 1.5 and 1.99 indicates that the model performs moderately well. An RPD between 2 and 2.5 indicates that the model performs well, and the model performs excellently when the RPD is higher than 2.5^33^.

3 $$ R_{C}^{2} = 1 - \frac{{\sum\nolimits_{i = 1}^{{n_{c} }} {\left[ {y_{act} (i) - y_{cal} (i)} \right]^{2} } }}{{\sum\nolimits_{i = 1}^{{n_{c} }} {\left[ {y_{act} (i) - ymean(i)} \right]^{2} } }} $$

4 $$ R_{P}^{2} = 1 - \frac{{\sum\nolimits_{i = 1}^{{n_{p} }} {\left[ {y_{act} (i) - ypre(i)} \right]^{2} } }}{{\sum\nolimits_{i = 1}^{{n_{p} }} {\left[ {y_{act} (i) - ymean(i)} \right]^{2} } }} $$

5 $$ {\text{RMSEC}} = \sqrt {\frac{1}{{n_{C} }}\mathop \sum \limits_{i = 1}^{{n_{c} }} \left[ {y_{act} (i) - y_{cal} (i)} \right]^{2} } $$

6 $$ {\text{RMSEP}} = \sqrt {\frac{1}{{n_{p} }}\mathop \sum \limits_{i = 1}^{{n_{p} }} \left[ {y_{act} (i) - ypre(i)} \right]^{2} } $$

7 $$ {\text{RPD}} = \frac{{\text{SD}}}{{\text{RMSEP}}} $$

where *n*_c_ and *n*_p_ denote the number of samples in the calibration and prediction sets; *y*_*act*_ and *y*_*mean*_ denote the measured and mean values; *y*_*cal*_ and *y*_*pre*_ denote the predicted values in the calibration and prediction sets, respectively; and SD denotes the standard deviation of the measured values in the prediction set.

## Results and discussion

### Spectral characteristics

**Figure 3:**
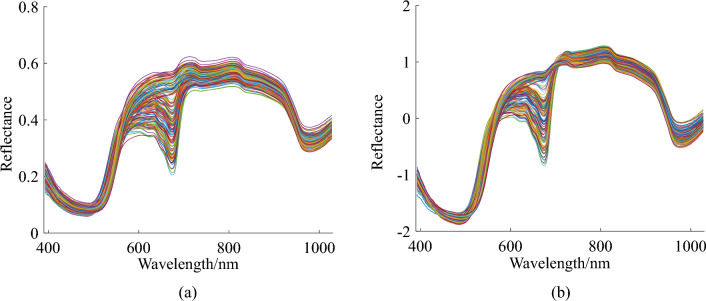
(**a**) Reflectance curves of raw spectra; (**b**) pretreated spectra of loquat samples.

The original and preprocessed (SNV) spectral curves are shown in Fig. 3. The spectra of the loquat samples showed the same tendency but with different reflection intensities. The preprocessed curves (Fig. 3b) were generally smoother than the original spectral curves (Fig. 3a), indicating a significant pretreatment effect. A clear absorption peak near 675 nm occurred, which correlated with the absorption of chlorophyll^34^. The more obvious absorption peak at approximately 980 nm may be attributed to the O–H chemical bond, which is related to water^35^.

### Statistical analysis of chemical concentration values

**Figure 4:**
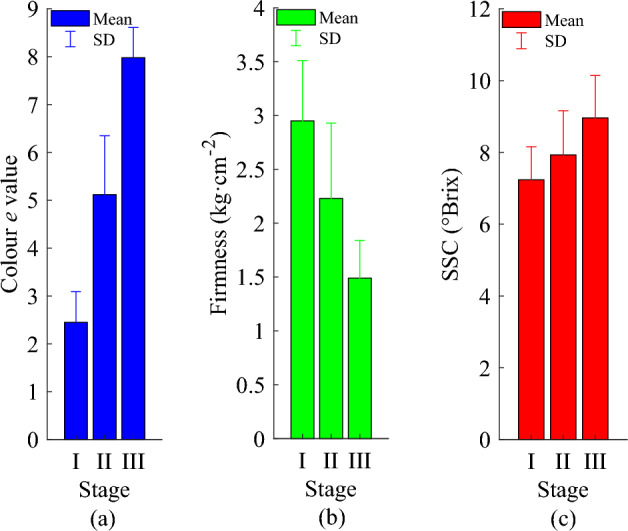
(**a**) Colour *e* value of loquat samples at different maturity stages; (**b**) firmness of loquat samples at different maturity stages; (**c**) SSC of loquat samples at different maturity stages.

Figure 4 shows colour *e* value, firmness, and SSC of loquat samples at three maturity stages; the data are shown as mean ± SD. There is an increasing trend for colour *e* value and SSC of loquats and a downward trend for firmness with maturity stages.

The SPXY algorithm^36^ was used to divide all the samples into calibration and prediction sets. The ratio of the calibration set to the prediction set was 3:1. Table 1 presents the calibration and prediction sets statistics for colour *e* value, firmness, and SSC. The range of values of the calibration set was wider than that of the prediction set, which indicated that the results for the calibration and prediction sets were reasonable and the selected modelling samples were highly representative.

**Table 1:** Statistics of colour *e* value, firmness and SSC of loquats.

Quality parameter	Calibration set				Prediction set			
	Num	Range	Mean	SD	Num	Range	Mean	SD
Color *e* value	105	1.17–9.08	4.60	2.13	35	1.27–8.88	5.55	2.43
Firmness/(kg/cm^2^)	105	0.85–4.06	2.39	0.81	35	0.93–3.26	1.80	0.63
SSC/(°Brix)	90	5.50–10.50	7.79	1.21	30	6.60–10.50	8.81	1.29

### Modelling based on full spectra

PLSR and PCR models were built up to assess the parameters of loquat quality using spectra preprocessed with SNV. The prediction results for the PLSR and PCR models are listed in Table 2.

**Table 2:** Performance of PLSR and PCR models for colour *e* value, firmness, and SSC.

Model	Quality parameter	LVs	Calibration set		Prediction set		
			*R*^2^_C_	RMSEC	*R*^2^_P_	RMSEP	RPD
PLSR	Color *e* value	12	0.96	0.42	0.96	0.49	4.97
	Firmness	12	0.86	0.30	0.82	0.27	2.39
	SSC	17	0.87	0.43	0.72	0.67	1.92
PCR	Color *e* value	22	0.96	0.43	0.95	0.51	4.76
	Firmness	16	0.81	0.35	0.80	0.28	2.29
	SSC	27	0.81	0.53	0.65	0.75	1.72

The prediction performances of the PLSR models for colour *e* value (*R*^2^_P_ = 0.96, RMSEP = 0.49, RPD = 4.97), firmness (*R*^2^_P_ = 0.82, RMSEP = 0.27, RPD = 2.39), and SSC (*R*^2^_P_ = 0.72, RMSEP = 0.67, RPD = 1.92) were better than those of the PCR models. This may be because the PLSR method has the advantage of considering both matrices, *x* (spectral matrix) and *y* (concentration matrix).

### Feature variables selection

#### Feature variables selected by CARS

When extracting the feature variables using CARS, the number of Monte Carlo sampling runs was set to 50, and the cross-validation of the group amount was set to five. The optimal feature variables was selected based on the minimal RMSECV, which corresponded to the sampling runs at 27, 23, and 28 for colour *e* value, firmness, and SSC, respectively. The selected variables were 20, 29, and 18 for colour *e* value, firmness, and SSC of loquats, respectively. Table 3 presents the detailed variables selected by CARS.

**Table 3:** Optimal variables for colour *e* value, firmness, and SSC selected by CARS, GA, and SPA.

Index	Methods	Number	Variables/nm
Color *e* value	CARS	20	397, 401, 404, 413, 418, 422, 432, 443, 498, 526, 541, 553, 621, 623, 641, 717, 750, 972, 974, 993
	GA	29	394, 397, 399, 401, 411, 413, 415, 420, 422, 425, 505, 507, 515, 517, 519, 522, 524, 526, 529, 536, 538, 541, 543, 546, 548, 551, 553, 555, 558
	SPA	3	551, 392, 577
Firmness	CARS	29	394, 399, 406, 408, 411, 413, 415, 555, 616, 619, 626, 641, 643, 678, 690, 693, 705, 707, 727, 730, 733, 743, 783, 785, 796, 974, 987, 1022, 1024
	GA	22	546, 548, 551, 553, 555, 558, 560, 675, 678, 680, 685, 688, 690, 693, 698, 700, 703, 801, 1006, 1019, 1022, 1024
	SPA	27	399, 406, 413, 415, 418, 422, 425, 429, 436, 439, 441, 448, 458, 493, 510, 526, 546, 577, 648, 678, 695, 745, 875, 914, 945, 1019, 1022
SSC	CARS	18	418, 425, 439, 488, 505, 695, 705, 720, 824, 883, 885, 888, 914, 919, 940, 961, 990, 1019
	GA	23	418, 420, 462, 465, 467, 474, 476, 479, 484, 507, 522, 524, 901, 904, 906, 917, 919, 982, 985, 987, 1016, 1019, 1022
	SPA	16	406, 408, 418, 425, 432, 443, 446, 450, 453, 481, 775, 867, 914, 937, 979, 1027

#### Feature variables selected by GA

The GA has a strong global optimisation ability. When extracting the feature variables using the GA, the population size, crossover probability, mutation probability, and the number of iterations were set to 30, 0.5, 0.01, and 100, respectively. The optimal combination of variables with the minimal RMSECV was viewed as the key variable to determine the parameters in the loquat. The number of corresponding feature variables set with the minimal RMSECV was 29, 22, and 23 for colour *e* value, firmness, and SSC in loquats, respectively. Table 3 lists the variables selected by the GA.

#### Feature variables selected by the SPA

For SPA, the number of variables was selected based on the minimum root mean square error (RMSE). Firstly, the RMSE decreases rapidly owing to the elimination of unimportant redundant variables. When the redundant information variable set of spectral information was minimal, the number of corresponding feature variables sets was 3, 27, and 16 for colour *e* value, firmness, and SSC in the loquat, respectively. Table 3 presents the detailed variables selected by the SPA.

### Modelling based on feature variables

The MLR, ELM, and BP models for predicting loquat quality were established based on these feature variables. The performances of the models are listed in Table 4.

**Table 4:** Prediction results of the MLR, ELM, and BP models.

Quality parameter	Model	Method	Band Num	Calibration set		Prediction set		
				*R*^2^_C_	RMSEC	*R*^2^_P_	RMSEP	RPD
Color *e* value	MLR	CARS	20	0.97	0.39	0.96	0.45	5.38
		GA	29	0.97	0.40	0.96	0.49	5.01
		SPA	3	0.92	0.62	0.98	0.37	6.60
	ELM	CARS	20	0.97	0.39	0.96	0.46	5.31
		GA	29	0.96	0.41	0.96	0.50	4.87
		SPA	3	0.93	0.57	0.97	0.40	6.08
	BP	CARS	20	0.96	0.42	0.96	0.51	4.78
		GA	29	0.96	0.44	0.94	0.58	4.20
		SPA	3	0.91	0.63	0.97	0.44	5.56
Firmness	MLR	CARS	29	0.90	0.26	0.87	0.23	2.81
		GA	22	0.85	0.31	0.79	0.29	2.20
		SPA	27	0.88	0.28	0.84	0.25	2.51
	ELM	CARS	29	0.85	0.31	0.83	0.26	2.43
		GA	22	0.83	0.34	0.75	0.31	2.04
		SPA	27	0.81	0.35	0.79	0.29	2.21
	BP	CARS	29	0.89	0.27	0.83	0.26	2.46
		GA	22	0.83	0.34	0.77	0.30	2.12
		SPA	27	0.84	0.32	0.82	0.27	2.39
SSC	MLR	CARS	18	0.88	0.41	0.84	0.51	2.54
		GA	23	0.76	0.59	0.59	0.82	1.58
		SPA	16	0.74	0.61	0.77	0.61	2.13
	ELM	CARS	18	0.86	0.45	0.76	0.63	2.06
		GA	23	0.74	0.61	0.62	0.78	1.65
		SPA	16	0.75	0.60	0.69	0.70	1.84
	BP	CARS	18	0.87	0.43	0.77	0.61	2.11
		GA	23	0.60	0.77	0.59	0.81	1.59
		SPA	16	0.72	0.64	0.57	0.84	1.54

As presented in Table 4, for colour *e* value, CARS was superior to the GA in setting the proper parameters. The models built based on the feature variables extracted by SPA exhibited the worst performance, with *R*^2^_C_ lower than *R*^2^_P_, which might be caused by under-fitting. The number of feature variables selected using CARS was 20, which represented 7.81% of the full spectrum. Compared with other models built based on feature variables selected by CARS, the MLR model built based on the feature variables extracted by CARS obtained a higher RPD and lower RMSEC and RMSEP. Compared with the models based on full wavelengths shown in Table 2, the prediction accuracy of MLR, ELM, and BP models based on feature variables selected by CARS and GA was enhanced. Especially, the CARS-MLR model achieved the best performance (*R*^2^_C_ = 0.97, RMSEC = 0.39, *R*^2^_P_ = 0.96, RMSEP = 0.45, and RPD = 5.38) in predicting colour *e* value.

For firmness, the CARS appeared to be superior to the SPA and GA regarding setting appropriate parameters. The number of feature variables selected by CARS was 29, which was 11.33% of the full spectrum. Compared with other models built based on the feature variables selected by CARS, the MLR model built based on the feature variables extracted by CARS obtained higher *R*^2^_C_, *R*^2^_P_, and RPD and lower RMSEC and RMSEP. Compared with the models based on full wavelengths shown in Table 2, the prediction accuracy of MLR, ELM, and BP models based on the feature variables selected by CARS and SPA was improved. Especially, the CARS-MLR model achieved the best performance (*R*^2^_C_ = 0.90, RMSEC = 0.26, *R*^2^_P_ = 0.87, RMSEP = 0.23, and RPD = 2.81) in predicting firmness.

For SSC, CARS appeared to be superior to the GA through the set of proper parameters. The accuracies of the SPA-ELM and SPA-BP models were lower than those of the CARS-ELM and CARS-BP models. The SPA-MLR model indicated the worst performance of *R*^2^_C_ lower than *R*^2^_P_, which might be caused by under-fitting. The number of feature variables selected by CARS was 18, which was 7.03% of the full spectrum. Compared with other models built based on the feature variables selected by CARS, the MLR model established based on the feature variables extracted by CARS obtained higher *R*^2^_C_, *R*^2^_P_, and RPD and lower RMSEC and RMSEP. Compared with the models based on full wavelengths shown in Table 2, the prediction accuracy of MLR, ELM, and BP models based on feature variables selected by CARS was improved. Especially, the CARS-MLR model achieved the best performance (*R*^2^_C_ = 0.88, RMSEC = 0.41, *R*^2^_P_ = 0.84, RMSEP = 0.51, and RPD = 2.54) in predicting SSC.

#### Modelling based on the optimal combinations of variables

**Figure 5:**
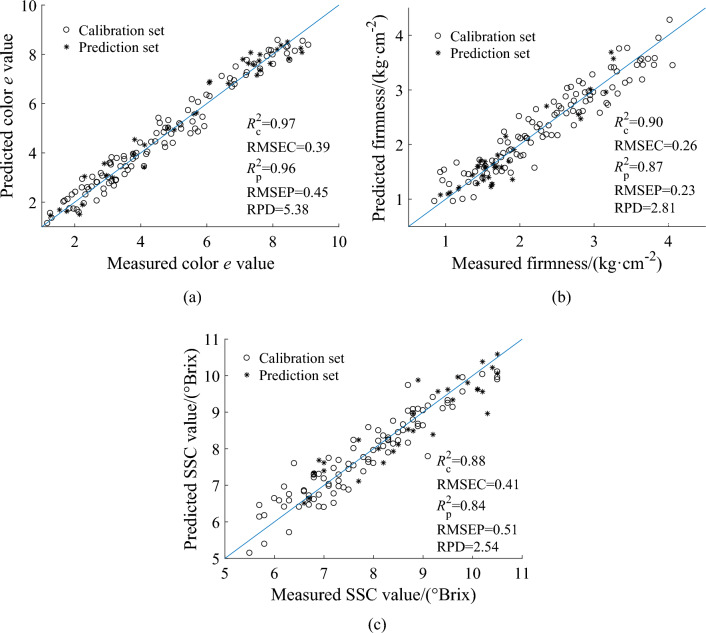
Scatter plots of the modelling results of the CARS-MLR model: (**a**) prediction results of colour *e* value; (**b**) prediction results of firmness; (**c**) prediction results of SSC.

MLR models using optimal feature variables selected by CARS were established to predict the quality of the loquats regarding colour *e* value, firmness, and SSC. The scatter plots of the actual measured and predicted values are shown in Fig. 5.

Figure 5 shows that the prediction errors of the three quality parameters were all small, and most of the data points were distributed near the fitting line, which indicates that the CARS-MLR model can predict loquat quality (colour *e* value, firmness, and SSC) very well.

The optimal CARS-MLR prediction model formulae for colour *e* value, firmness, and SSC of loquats are as follows:

8 $$ \begin{aligned} Y_{colour} {\kern 1pt}_{{\text{e}}} {\kern 1pt}_{value} =\,  & 22.89 - 8.82\lambda_{397} + 12.37\lambda_{401} - 22.02\lambda_{404} + 28.37\lambda_{413} + 2.09\lambda_{418} \\ & + 20.83\lambda_{422} - 11.90\lambda_{432} - 24.50\lambda_{443} + 23.75\lambda_{498} - 17.75\lambda_{526} + 20.45\lambda_{541} \\ & - 16.34\lambda_{553} - 0.04\lambda_{621} + 21.10\lambda_{623} - 8.06\lambda_{641} - 15.07\lambda_{717} + 14.64\lambda_{750} \\ & + 23.98\lambda_{972} - 0.96\lambda_{974} - 18.62\lambda_{993} \\ \end{aligned} $$

9 $$ \begin{aligned} Y_{{F{\text{irmness}}}} = & \, 13.36 - 11.57\lambda_{394} + 19.69\lambda_{399} - 14.56\lambda_{406} - 21.74\lambda_{408} + 11.40\lambda_{411} \\ & + 21.95\lambda_{413} - 1.14\lambda_{415} + 5.08\lambda_{555} - 72.32\lambda_{616} + 98.67\lambda_{619} - 48.77\lambda_{626} \\ & + 8.86\lambda_{641} + 5.32\lambda_{643} - 1.30\lambda_{678} - 31.62\lambda_{690} + 53.08\lambda_{693} + 22.26\lambda_{705} \\ & - 61.38\lambda_{707} + 100.76\lambda_{727} - 95.06\lambda_{730} + 48.17\lambda_{733} - 11.88\lambda_{743} - 9.22\lambda_{783} \\ & - 23.11\lambda_{785} + 10.39\lambda_{796} - 15.85\lambda_{974} + 1.51\lambda_{987} + 12.89\lambda_{1022} + 7.36\lambda_{1024} \\ \end{aligned} $$

10 $$ \begin{aligned} Y_{SSC} =\,  & 36.33 - 76.42\lambda_{418} + 84.97\lambda_{425} + 26.85\lambda_{439} - 48.79\lambda_{488} + 30.30\lambda_{505} \\ & - 21.61\lambda_{695} + 77.22\lambda_{705} - 67.04\lambda_{720} - 29.14\lambda_{824} - 31.90\lambda_{883} + 222.32\lambda_{885} \\ & + 61.35\lambda_{888} - 176.46\lambda_{914} - 122.60\lambda_{919} + 72.51\lambda_{940} + 68.45\lambda_{961} - 134.16\lambda_{990} \\ & + 81.12\lambda_{1019} \\ \end{aligned} $$

where *Y*_*colour e value*_, *Y*_*Firmness*_, and *Y*_*SSC*_ represent the predicted values for colour *e* value, firmness, and SSC, respectively. *λ*_*i*_ denotes the reflectance at the feature wavelength, where the subscript *i* indicates the wavelength (nm).

### Visualised distribution of quality parameters

**Figure 6:**
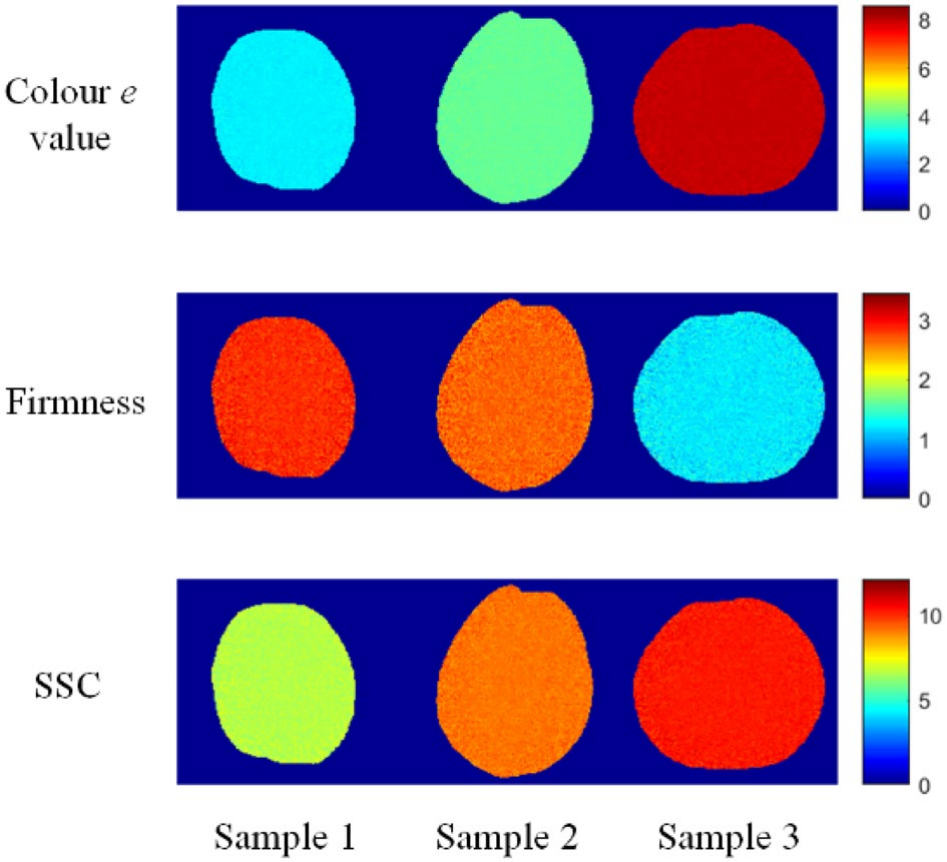
Prediction maps for colour *e* value, firmness, and SSC in different loquat samples.

A feature of the HSI technique is that information can be gathered from each pixel of the test sample^37^. The information extracted from the hyperspectral images was used to generate visualisation distribution maps of the reference values (colour *e* value, firmness, and SSC), which enabled visualisation of the differences in the reference values between the samples^38^. Due to the approximately spherical shape of loquat fruit, the spectra of different pixels within the same fruit region may exhibit significant differences, potentially leading to poor imaging results. One specific application of loquat fruit detection is to evaluate the overall fruit quality, with secondary emphasis on expressing local characteristics. Building upon this fact, the deviation between the pixel values and the mean spectrum is compressed, and the sum of the compressed deviation and the mean spectrum is employed as the input variable^17^. In this study, the optimal CARS-MLR models were used to predict the quality parameter content of each pixel in loquat^39^. Figure 6 shows the intuitive distribution of colour *e* value, firmness, and SSC for samples 1, 2, and 3, respectively. The samples 1, 2, and 3 correspond to maturity stages I, II, and III, respectively.

As shown in Fig. 6, colour *e* value and SSC gradually increased with the different maturity stages, while firmness gradually decreased with the different maturity stages. And there were significant visual differences between the different samples. Therefore, the distribution map is useful for online monitoring of loquat quality.

### Maturity stage classification

A total of 249 samples were used for classifying loquat maturity, with 60 samples in stage I, 150 in stage II, and 39 in stage III. The Kennard–Stone algorithm was applied to partition the samples from each stage into calibration and prediction sets at a ratio of 2:1, resulting in 166 and 83 samples in the calibration and prediction sets, respectively. The PLS-DA, simplified K nearest neighbor (SKNN), and SVM models were applied to discriminate the maturity stages of loquats. The discrimination results are listed in Table 5.

**Table 5:** Prediction results of maturity stages of loquat by PLS-DA, SKNN, and SVM models.

Model	Calibration set			Prediction set			Total accuracy/%
	Num	Error	Accuracy/%	Num	Error	Accuracy/%	
PLS-DA	166	3	98.19	83	2	97.59	97.99
SKNN	166	14	91.57	83	2	97.59	93.57
SVM	166	9	94.58	83	2	97.59	95.58

**Figure 7:**
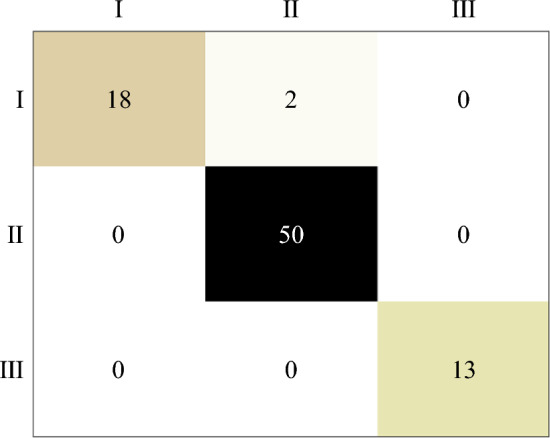
Confusion matrix of prediction set.

As presented in Table 5, the PLS-DA model had a higher discrimination accuracy in the calibration set than the SKNN and SVM models. The three models had the same discrimination accuracy (97.59%) for the prediction set. Figure 7 shows the confusion matrix of the prediction set, in which two samples from Stage I were incorrectly identified as Stage II in each of the PLS-DA, SKNN, and SVM models. The results illustrated that the PLS-DA model had the best performance in discriminating loquat maturity.

## Conclusions

In this study, hyperspectral imaging technology was used to detect and visualise loquat quality and discriminate maturity. The main findings of this study are as follows.

1. Hyperspectral imaging coupled with chemometric algorithms is a feasible method for assessing loquat quality. Comparing full spectra models (PLSR and PCR) with simplified models (MLR, ELM, and BP network) based on feature variables selected by three effective variables selection algorithms (CARS, GA, and SPA), the CARS-MLR models with the optimal prediction performance were obtained for colour *e* value (*R*^2^_P_ = 0.96, RMSEP = 0.45, RPD = 5.38), firmness (*R*^2^_P_ = 0.87, RMSEP = 0.23, RPD = 2.81), and SSC (*R*^2^_P_ = 0.84, RMSEP = 0.51, RPD = 2.54), respectively.
2. The optimal prediction model combined with pseudo-colour technology could visualise the quality parameter distribution of loquats. The maps show that the distribution of the quality parameters essentially corresponded to the actual situation, and the content of the same quality parameters was significantly different between the loquat samples.
3. Hyperspectral imaging combined with pattern recognition can be used to evaluate loquat maturity. On comparison of the three maturity classification models (PLS-DA, SKNN, and SVM models), the PLS-DA model showed the best performance, with classification accuracies of 98.19% and 97.99% for calibration and prediction sets, respectively.

This study indicates that hyperspectral imaging technology can be used to non-destructively and rapidly determine loquat quality and maturity, providing a theoretical basis for the development of instruments in the future.

## Data Availability

The data that support the findings of this study are available from the corresponding author upon reasonable request.
